# Acute Myocardial Infarction Due to Eltrombopag Therapy in a Patient with Immune Thrombocytopenic Purpura

**DOI:** 10.4274/tjh.2016.0169

**Published:** 2017-03-01

**Authors:** Sena Sert, Hasan Özdil, Murat Sünbül

**Affiliations:** 1 Marmara University Faculty of Medicine, Department of Cardiology, İstanbul, Turkey

**Keywords:** Acute myocardial infarction, Immune thrombocytopenic purpura, Eltrombopag

## TO THE EDITOR,

Immune thrombocytopenic purpura (ITP) is an autoimmune disease characterized by anti-platelet antibody-mediated platelet destruction and anti-megakaryocyte antibody-mediated impairment of platelet production, which may cause bleeding [1]. Coexistence of ITP and coronary artery disease (CAD) is rare. Patients with ITP have increased risk of thrombosis and atherosclerosis associated with larger platelets more adhesive to vascular surfaces, direct endothelial damage [2], and negative effects of therapy with steroids [3] or intravenous immunoglobulin [4]. We present here a 61-year-old male patient who was diagnosed with ITP and presented with acute myocardial infarction while undergoing eltrombopag therapy.

**Figure 1:**
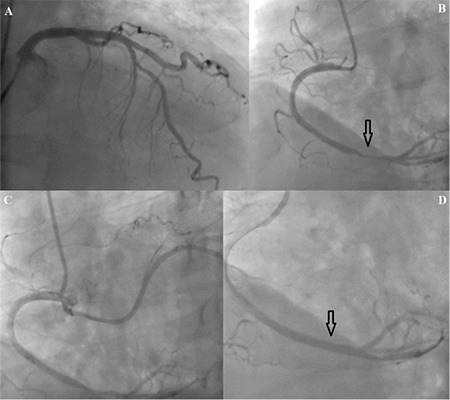
A) Non-critical plaque on the proximal and middle portion of the left anterior descending artery, B) arrow shows sub-total occlusion at the distal portion of the right coronary artery, C) circumflex coronary artery originates from the right aortic root, D) bare metal stent (3.0x20 mm) was implanted at the lesion site and post-dilatation was performed with a noncompliant balloon (3.5x15 mm).

A 61-year-old man was admitted to our emergency room with typical chest pain lasting for last 3 days. He had been diagnosed with ITP 5 years ago. His medical history was remarkable for splenectomy 6 months after the diagnosis of ITP. He was in remission for 4 years after the splenectomy and he was not on any medication for 5 years. Four months before, during a routine check-up, relapse of disease had been noticed. Steroid therapy was initiated after relapse and administered with a tapering dosage for 3 months. The clinician did not observe adequate increase in the amount of platelets; therefore, eltrombopag (1x50 mg tablet) was initiated as a newline therapy 1 month ago. In the first 3 weeks, the platelet count did not increase adequately (platelets were about 13,000/mL), but in the last week before his admission to the emergency room his platelet count escalated to about 105,000/mL. The patient was admitted to our emergency room with typical chest pain. His baseline cardiovascular risk factors, among smoking, hyperlipidemia, hypertension, diabetes mellitus, and family history, were not remarkable. The patient was not on any medication apart from eltrombopag therapy. On his admission, electrocardiography showed ST segment elevation in leads DII-III-AVF and V5-6 with pathological Q waves, which gave rise to consideration of sub-acute inferolateral myocardial infarction. Primary percutaneous coronary intervention (PCI) was performed immediately. Coronary angiography demonstrated the anomalous origin of the coronary artery. The circumflex coronary artery (CX) originated from the right aortic root. There was plaque on the proximal and middle portion of the left anterior descending artery and proximal portion of the CX, and subtotal occlusion at the distal portion of the right coronary artery (RCA). A bare metal stent was implanted at the lesion site and post-dilatation was performed (Figures 1A-1D). After PCI, thrombolysis in myocardial infarction grade 3 flow was obtained as an optimal angiographic result in the RCA. Platelet counts were assessed daily and showed a stable trend. At the suggestion of the hematology department, the eltrombopag therapy was stopped. The patient was examined for an underlying hypercoagulable state. His homocysteine level was within normal limits. Antinuclear antibodies, antiphospholipid and anticardiolipin antibodies, lupus-like anticoagulant, and mutations of factor V Leiden were negative. The patient was discharged on the 5^th^ day with a platelet count of 125,000/mL, with advice to continue dual anti-platelet therapy (acetylsalicylic acid 100 mg and clopidogrel 75 mg). There was no relapse for ITP during the 1-year follow-up period (Table 1).

Eltrombopag is an orally available, small, non-peptide organic molecule that enhances platelet production by binding to and activating c-Mpl, the thrombopoietin receptor, on megakaryocytes and their progenitors [5]. The main issue in our case is that, as we mentioned the importance of evaluating risk factors, our patient had no risk factors for CAD and we recognized the coincidence between acute coronary syndrome and the beginning of a new agent of thrombopoietin receptor agonist (TPO-A) therapy. TPO-A therapy has important side effects including thromboembolic events [6,7,8]. A recent study demonstrated that an important percentage of ITP patients undergoing eltrombopag therapy achieve complete response after cessation of the therapy. There is no reliable marker for predicting this response so far [9].

Coexistence of ITP and CAD presents complex problems. The crucial point in handling these problems is a balance between hemorrhagic risk and prevention of thrombotic events. Although eltrombopag is more effective in the treatment of patients with ITP, clinicians should pay more attention to side effects including thrombotic events, as we demonstrated in our case report.

## Figures and Tables

**Table 1:**
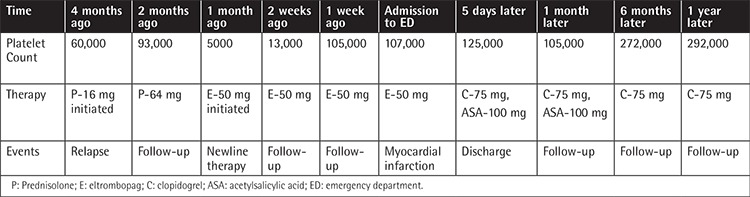
The relationship between medical treatments and platelet counts during follow-up periods.

## References

[1] Cines DB, Bussel JB, Liebman HA, Luning Prak ET (2009). The ITP syndrome: pathogenic and clinical diversity. Blood.

[2] Fruchter O, Blich M, Jacob G (2002). Fatal acute myocardial infarction during severe thrombocytopenia in a patient with idiopathic thrombocytopenic purpura. Am J Med Sci.

[3] Paolini R, Zamboni S, Ramazzina E, Zampieri P, Cella G (1999). Idiopathic thrombocytopenic purpura treated with steroid therapy does not prevent acute myocardial infarction: a case report. Blood Coagul Fibrinolysis.

[4] Elkayam O, Paran D, Milo R, Davidovitz Y, Almoznino-Sarafian D, Zeltser D, Yaron M, Caspi D (2000). Acute myocardial infarction associated with high dose intravenous immunoglobulin infusion for autoimmune disorders. A study of four cases. Ann Rheum Dis.

[5] Erickson-Miller CL, DeLorme E, Tian SS, Hopson CB, Landis AJ, Valoret EI, Sellers TS, Rosen J, Miller SG, Luengo JI, Duffy KJ, Jenkins JM (2009). Preclinical activity of eltrombopag (SB-497115), an oral, nonpeptide thrombopoietin receptor agonist. Stem Cells.

[6] Saleh MN, Bussel JB, Cheng G, Meyer O, Bailey CK, Arning M, Brainsky A, EXTEND Study Group (2013). Safety and efficacy of eltrombopag for treatment of chronic immune thrombocytopenia: results of the long-term, open-label EXTEND study. Blood.

[7] Hassn AMF, Al-Fallouji MA, Ouf TI, Saad R (2000). Portal vein thrombosis following splenectomy. Br J Surg.

[8] Harker LA, Hunt P, Marzec UM, Kelly AB, Tomer A, Hanson SR, Stead RB (1996). Regulation of platelet production and function by megakaryocyte growth and development factor in nonhuman primates. Blood.

[9] González-López TJ, Pascual C, Álvarez-Román MT, Fernández-Fuertes F, Sánchez-González B, Caparrós I, Jarque I, Mingot-Castellano ME, Hernández-Rivas JA, Martín-Salces M, Solán L, Beneit P, Jiménez R, Bernat S, Andrade MM, Cortés M, Cortti MJ, Pérez-Crespo S, Gómez-Núñez M, Olivera PE, Pérez-Rus G, Martínez-Robles V, Alonso R, Fernández-Rodríguez A, Arratibel MC, Perera M, Fernández-Miñano C, Fuertes-Palacio MA, Vázquez-Paganini JA, Gutierrez-Jomarrón I, Valcarce I, Cabo E, Sainz A, Fisac R, Aguilar C, Paz Martínez-Badas M, Peñarrubia MJ, Calbacho M, Cos C, González-Silva M, Coria E, Alonso A, Casaus A, Luaña A, Galán P, Fernández-Canal C, Garcia-Frade J, González-Porras JR (2015). Successful discontinuation of eltrombopag after complete remission in patients with primary immune thrombocytopenia. Am J Hematol.

